# Genetic Insights and Molecular Breeding Approaches for Downy Mildew Resistance in Cucumber (*Cucumis sativus* L.): Current Progress and Future Prospects

**DOI:** 10.3390/ijms252312726

**Published:** 2024-11-27

**Authors:** Ewa Mirzwa-Mróz, Bartłomiej Zieniuk, Zhimin Yin, Magdalena Pawełkowicz

**Affiliations:** 1 Division of Plant Pathology, Department of Plant Protection, Institute of Horticultural Sciences, Warsaw University of Life Sciences-SGGW, 159 Nowoursynowska Str., 02-776 Warsaw, Poland; ewa_mirzwa-mroz@sggw.edu.pl; 2 Department of Chemistry, Institute of Food Sciences, Warsaw University of Life Sciences SGGW, 159C Nowoursynowska Str., 02-776 Warsaw, Poland; bartlomiej_zieniuk@sggw.edu.pl; 3 Plant Breeding and Acclimatization Institute–National Research Institute in Radzików, Młochów Division, Department of Potato Genetics and Parental Lines, 19 Platanowa Str., 05-831 Młochów, Poland; z.yin@ihar.edu.pl; 4 Department of Plant Genetics, Breeding and Biotechnology, Institute of Biology, Warsaw University of Life Sciences-SGGW, 159 Nowoursynowska Str., 02-776 Warsaw, Poland

**Keywords:** cucurbit downy mildew, *Pseudoperonospora cubensis*, disease control, Cucurbitaceae

## Abstract

Cucurbit downy mildew, caused by *Pseudoperonospora cubensis*, is a devastating disease in cucumbers that leads to significant yield losses in many cucurbit-growing regions worldwide. Developing resistant cucumber varieties is a sustainable approach to managing this disease, especially given the limitations of chemical control and the evolving nature of pathogens. This article reviews the genetic basis of downy mildew resistance in cucumbers, emphasizing key resistance (R) genes and quantitative trait loci (QTLs) that have been mapped. Recent advances in molecular breeding tools, including marker-assisted selection (MAS), genomic selection (GS), and CRISPR/Cas9 genome editing, have accelerated the development of resistant cultivars. This review also explores the role of transcriptomics, genomics, and other ‘omics’ technologies in unraveling the molecular mechanisms behind resistance and offers insights into the future of breeding strategies aimed at long-term disease management. Management strategies for cucurbit downy mildew are discussed, along with the potential impacts of climate change on the occurrence and severity of downy mildew, highlighting how changing environmental conditions may influence disease dynamics. Integrating these advanced genetic approaches with traditional breeding promises to accelerate the development of downy mildew-resistant cucumber varieties, contributing to the sustainability and resilience of cucumber production.

## 1. Introduction

The Cucurbitaceae family consists of 825 species in 118 genera, of which approximately 150 are extensively grown and 30 are crucial for global food production. The cultivation of cucurbits for food purposes commenced over 3000 years ago in Western Asia [1,2]. *Cucumis sativus* L., also known as cucumber, had a global production of 94 million metric tons in 2022. This was harvested from 2.2 million hectares, with China, Turkey, Russia, Mexico, and Uzbekistan being the top five producing countries, representing 87% of the world’s production [3]. Cucumbers are believed to be native to Asia, primarily Nepal and India [4]. The wild ancestor of present-day cucumbers, the bitter-fruited form, *C. sativus* var. *hardwickii*, has been grown in this region for 3000 years. The cucumber was introduced to Europe during the medieval period. According to Paris et al. [5], cucumbers were not present in Roman times up to 500 CE, appeared in France in the 9th century, in the United Kingdom in the 14th century, and in North America by the mid-16th century [6].

Cucumbers serve both culinary and therapeutic roles in various cultures. They are primarily known for their crisp texture and high water content, comprising over 90% water. This makes them a popular ingredient in salads, eaten fresh, fermented, or pickled [6]. However, they are low in flavor and nutrition, with only 65 kJ (16 kcal) of energy, 0.65 g of proteins, 0.11 g of lipids, and 3.63 g of carbohydrates (1.67 of sugars) per 100 g [7]. Cucumbers also contain bioactive phytochemicals like cucurbitacins and flavonoids and have various medicinal uses, such as detoxifying the body, nourishing the skin, soothing irritations, and alleviating sunburn pain [8,9].

Considering research activity on this plant species, *C. sativus* is recognized as a model organism for exploring the intricacies of plant sex determination [10], and, most importantly, it was the very first plant within the Cucurbitaceae family whose genome was sequenced [11,12,13].

Unfortunately, cucumbers can be affected by a range of diseases that may impact their growth and yield. The most important are downy mildew, powdery mildew, and fusarium wilt [14]. Cucurbit downy mildew significantly impacts cucumber cultivation, leading to yield losses. As a result, it is recognized as the most destructive disease affecting cucumbers that has been reported in over 70 countries [15].

This review focuses on cucurbit downy mildew’s impact on cucumber cultivation, highlighting resistance (R) genes and QTLs identified through mapping studies. Advances in molecular breeding, such as marker-assisted selection (MAS), genomic selection (GS), and CRISPR/Cas9, have accelerated the development of resistant cultivars. The role of transcriptomics, genomics, and other ‘omics’ technologies in understanding resistance mechanisms and shaping sustainable breeding strategies is also discussed.

## 2. Downy Mildew Disease in Cucumbers

### 2.1. Geographic Distribution, Host Range, and Economic Importance

Cucurbit downy mildew is caused by the oomycete *Pseudoperonospora cubensis* (Berk. et M.A. Curtis) Rostovzev. This disease is widely distributed in many regions across the world. The development of this disease is primarily influenced by environmental conditions, particularly humid and warm climates [15,16]. It affects cucurbit crops in North and South America, Europe, Africa, and East and Southeast Asia, particularly in regions like the U.S., Brazil, India, and China. Airborne spores aid its spread across over 70 countries, with outbreaks often coinciding with monsoon seasons or tropical conditions [17,18,19,20]. Its global range spans over 70 countries for cucumbers, with airborne spores contributing to its spread in regions with favorable climates for disease progression [21].

*P. cubensis* has an extensive host range and can infect over 60 different plant species in the Cucurbitaceae family. The most frequently affected are cucumbers (*C. sativus* L.), melons (*C. melo* L.), common summer and winter squashes (*Cucurbita pepo* L.), gourds (*Cucurbita maxima* Duchesne and *C. moschata* Duchesne), and watermelon (*Citrullus lanatus* (Thunb.) Matsum. et Nakai) [22,23,24,25,26,27].

Cucurbit downy mildew is particularly destructive to cucurbits, including cucumbers, cantaloupes, pumpkins, and watermelons, and is known for its rapid progression and significant economic impact [15,28,29]. Since 1985, cucurbit downy mildew has been the most destructive disease in cucumbers in Central [30] and Northern [31] Europe. Disease outbreaks can be responsible for annual yield losses of up to 80% [30,32]. According to Holmes et al. [33], even up to 100% cucumber yield reduction is possible when fungicides are not used. If fungicides are applied 1 week after symptoms appear, yield is reduced by approximately 50%. In the United States, disease resurgence due to the loss of cucumber cultivar resistance has led to severe epidemics [34]. In 2004, the eastern United States noted a $16 million USD loss caused by cucurbit downy mildew [22]. In 2017, loss per cucurbit crop cycle caused by cucurbit downy mildew ranged from $50 to $1425 per acre [35].

### 2.2. Taxonomy of Pathogen, Symptoms and Signs

*P. cubensis* belongs to the kingdom Chromista, phylum Oomycota, order Peronosporales, family Peronosporaceae, and genus *Pseudoperonospora* [23,36]. It was first described in Cuba in 1868 [37].

While Choi et al. [38] suggest *Pseudoperonospora humuli* may be a synonym due to morphological and ITS sequence similarities, molecular studies and host range data by Mitchell et al. support their distinction [39,40].

**Figure 1:**
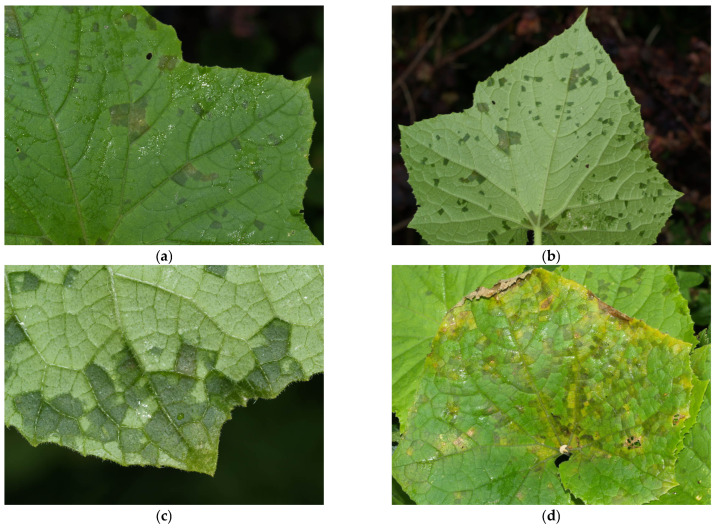

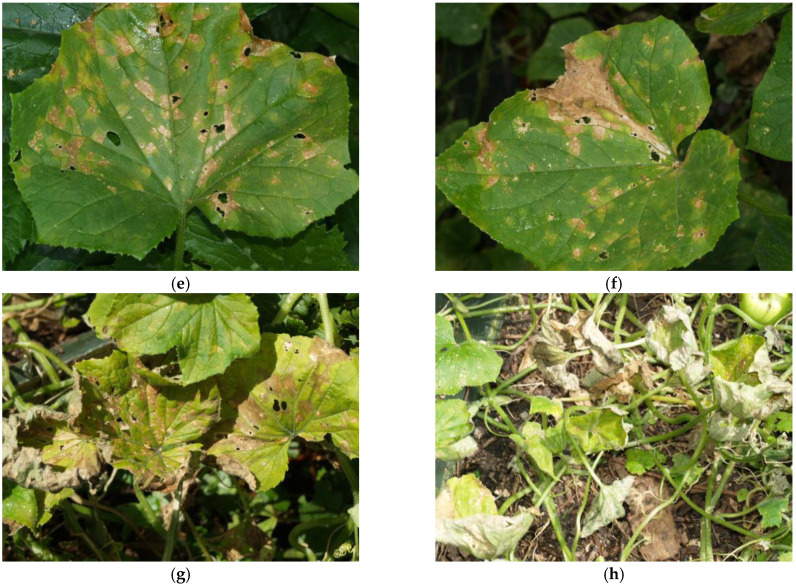
Symptoms of cucurbit downy mildew on cucumber: (**a**) chlorotic lesions on the upper leaf surface restricted by leaf veins, (**b**) spots soaked with water on the underside of the leaf, (**c**) magnification of spots soaked with water, (**d**) chlorotic and yellow lesions on the upper leaf surface, (**e**) brown, necrotic spots, (**f**) merging necrotic spots, (**g**) upward leaf curling, and (**h**) dying plants (Photos C. Zamorski).

The symptoms of cucurbit downy mildew on cucumber vary depending on the genotype (cultivar) of the plant [17,23]. They can appear on any stage of the plant’s development, including the leaves. It is important to note that *P. cubensis* does not infect the fruit directly [41]. The first symptoms of downy mildew appear on the leaves as small, angular, chlorotic spots on the upper leaf surface, which are restricted by leaf veins (Figure 1a) [15,23,29]. Early in the morning, when there is dew, or during rainfall, the spots are soaked with water (Figure 1b,c) [12,41]. This makes them similar to the spots caused by the bacteria *Pseudomonas syringae* pv. *lachrymans* [42]. As the disease progresses, spots may enlarge and turn yellow, then brown, leading to necrosis (Figure 1d,e) [15,22,23]. Next, the spots coalesce to produce larger spots that finally cover the entire leaf, often resulting in upward leaf curling (Figure 1f,g) and, under heavy infection, death of the whole plant (Figure 1h) [23]. In severe cases, extensive defoliation can occur, leading to reduced fruit yield and quality, as well as increased exposure of fruit to sunscald [15,22,23,43].

**Figure 2:**
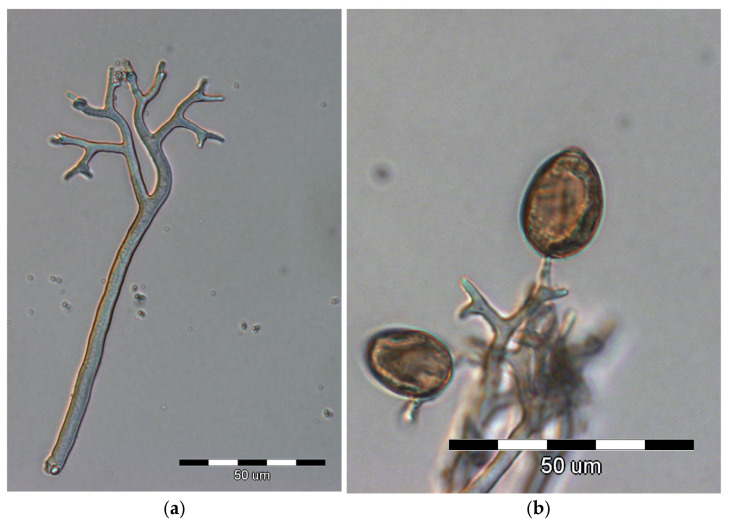
Sporulation of *P. cubensis* on the underside of the leaf: (**a**) sporangiophore and (**b**) dark sporangia (Photos E. Mirzwa-Mróz).

The most distinctive feature is the grayish to purplish downy coating on the undersides of leaves, which consists of sporangiophores and sporangia produced by the pathogen (Figure 2a,b). Sporangiophores (Figure 2a) are colorless and range from 180 to 400 µm in length, straight 5–7 µm wide. Branches are monopodial or dichotomous. The ultimate branchlets feature subacute apices. Sporangia (Figure 2b) are pale grayish to olivaceous-purple, and ovoid to ellipsoidal (20–40 × 14–25 µm) with pedicel [38]. The presence of a downy coating on the underside of the spot is crucial in disease diagnosis.

### 2.3. Biology of the Pathogen and Its Life Cycle

**Figure 3:**
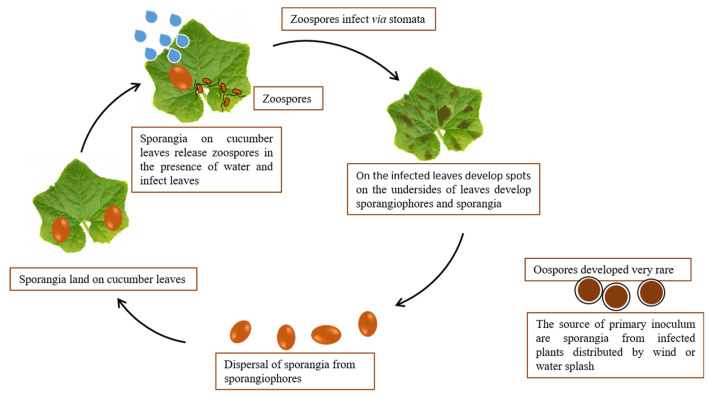
Life cycle of *P. cubensis*.

*P. cubensis* is an obligate plant pathogen (biotroph) that cannot survive outside its host plant except as oospores [15,23,24]. The role of oospores of these pathogens in the life cycle is unknown because sexual reproduction is rare [22,23]. The pathogen cannot overwinter in geographical locations with freezing winter temperatures. It can overwinter in areas with mild winters where cucurbits are present [15] or in greenhouses [16]. The sources of primary inoculum are sporangia from infected plants distributed by wind or water splash [23]. Changes in temperature and humidity influence the detachment and dispersal of sporangia from sporangiophores. After sporangia land on cucumber leaves, they release from 5 to 15 biflagellate zoospores in the presence of water (e.g., rain or dew). The zoospores move to stomatal apertures and lose their flagella and encyst [44]. From the encysted zoospores grow germ tubes that produce appressoria. The last ones form penetration hyphae and penetrate the leaf through stomata [15,22,23] (Figure 3).

The pathogen develops under cool, moist conditions, with optimal temperatures for infection around 15 °C (59 °F) and 2 h of leaf wetness [15,44]. Downy mildew is favored by high humidity and leaf wetness, which can occur from morning dew or rainfall. The disease typically appears during late summer to early fall, particularly in regions with warm, humid climates [15,22,23]. The incubation period can range from 4 to 12 days and depends on inoculum concentration, photoperiod, temperature, and leaf wetness duration [15,16,45].

## 3. Resistance of Cucumber to Cucurbit Downy Mildew—Molecular and Genetics Breeding Approaches

The genetic basis of downy mildew resistance in cucumbers (*C. sativus*) has been studied extensively due to the serious threat posed by the pathogen *P. cubensis*. In order to breed resistant varieties and effectively control the disease, a better understanding of its genetic basis is essential. Research has consistently shown that downy mildew resistance in cucumber is recessively inherited and involves multiple genes. This finding is strongly supported by the results of many studies, as highlighted in the following sources [46,47,48,49,50,51].

Resistance to downy mildew in cucumbers is primarily controlled by a recessive gene known as *dm-1*, which was first identified in the Indian cucumber accession PI 197087. This gene has been widely utilized in breeding programs to enhance resistance in commercial cultivars [15,52]. Additionally, multiple recessive genes contribute to resistance, indicating a polygenic inheritance pattern. For instance, cucumber accessions such as PI 197088 and others have been shown to possess several quantitative trait loci (QTLs) that confer varying levels of resistance [14,53,54].

In recent years, numerous QTLs linked to downy mildew resistance have been identified across various cucumber varieties. These discoveries have been made using molecular markers such as Sequence Characterized Amplified Regions (SCAR), Simple Sequence Repeats (SSR), and Single Nucleotide Polymorphisms (SNP).

A genetic linkage map developed with 66 polymorphic SSR markers led to the identification of 14 QTLs, which were assessed for downy mildew resistance in cotyledons and true leaves following inoculation. One of these QTLs, LG5.1, located between markers SSR03943 and SSR19172, was consistently identified at all stages of leaf development [55]. Another study used a more extensive linkage map consisting of 328 SSR and SNP markers to reveal significant QTLs, such as dm4.1 and dm5.1, which explained 15–30% of the phenotypic variance related to downy mildew resistance. These findings were confirmed across four different environments (US2013, US2014, IT2013, and NL2013) [56]. In seven independent experiments, five downy mildew-related QTLs were mapped to chromosomes 1, 3, 4, and 5, with dm4.1 being the most impactful, accounting for 27% of the phenotypic variance, and consistently observed in indoor trials [57]. The detection of downy mildew resistance QTLs has involved diverse evaluation strategies, including different plant organs (cotyledons and true leaves), developmental stages (from seedlings to adult plants), and assessment criteria such as lesion size and sporulation extent. Among the nine QTLs detected, dm1.1 had the most substantial effect on resistance [48].

In addition to traditional QTL mapping, advanced techniques like bulked segregant analysis (BSA), next-generation sequencing (NGS), and genome-wide association studies (GWAS) have expedited research on downy mildew resistance. BSA and GWAS pinpoint genomic regions of interest, while NGS allows for detailed sequencing of these regions to identify specific mutations or alleles associated with traits. For example, the combination of BSA and NGS identified five major QTLs (*dm2.2*, *dm4.1*, *dm5.1*, *dm5.2*, and *dm6.1*), with *dm2.2* showing the most significant effect [58]. In addition, GWAS of 97 cucumber lines revealed 18 QTLs, 6 of which (*dmG1.4*, *dmG4.1*, *dmG4.3*, *dmG5.2*, *dmG7.1*, and *dmG7.2*) were consistently associated with stable downy mildew resistance [59]. Cucumber lines, including PI 197085, PI 197088, and WI 7120 (PI 330628), have been extensively used in quantitative trait locus (QTL) mapping studies, providing insights into the stable genetic architecture underlying downy mildew resistance in different germplasm sources. Remarkably, the dm5.1 and dm5.2 QTLs have been consistently identified in resistant lines, highlighting their importance for resistance breeding [60]. In widely used disease-resistant cucumber cultivars, new QTLs have also been identified. For example, Berg et al. [54] identified three sub-QTLs within QTL DM4.1 on chromosome 4 in PI 197087: *DM4.1.1*, associated with pathogen-induced necrosis; DM4.1.2, associated with improving sporulation resistance; and *DM4.1.3*, with recessive effects on chlorosis and sporulation. Overall, the identification of downy mildew-associated QTLs varies depending on the cucumber germplasm, plant tissue, and developmental stage being analyzed.

Several candidate genes and proteins have been identified using transcriptome profiling, proteome analysis, and fine mapping. Transcriptome analyses comparing resistant and susceptible cucumber genotypes identified numerous differentially expressed genes (DEGs) associated with a range of defense mechanisms. These mechanisms include hormone signaling, nutrient regulation, pathogen recognition, signal transduction, reactive oxygen species (ROS) production, lignin biosynthesis, protein metabolism, and transcriptional regulation [61,62,63]. Notably, five key genes were implicated in the defense response against downy mildew in cucumber. *Csa5G139760* encodes an acidic chitin endonuclease, facilitating pathogen degradation. *Csa6G080320* codes for a leucine-rich repeat (LRR) and transmembrane domain kinase, crucial for signal transduction in immune responses. *Csa5G471600* is a retroviral receptor-like protein potentially involved in pathogen recognition, while *Csa5G544050* and *Csa5G564290* encode RNA-dependent RNA polymerases, which may contribute to post-transcriptional gene silencing as part of antiviral defenses [63].

Alongside these molecular findings, a large number of QTLs associated with downy mildew resistance have been identified, including dm2.1, dm4.1, dm4.1.2, dm4.1.3, dmG2.1 and dmG7.1 [54,58,59]. These QTLs, together with precise molecular markers, are valuable tools for fine mapping and positional cloning. For example, Liu et al. [59] identified seven candidate genes for downy mildew resistance using GWAS, including *Csa1G575030* for *dmG1.4* and for *dmG2.1*, *Csa4G064680* for *dmG4.1*, *Csa7G004020* for *dmG7.1*, and *Csa5G606470* for *dmG5.2*, the latter encoding a WRKY transcription factor, and *Csa5M622830.1* encoding a GATA transcription factor that may inhibit nutrient supply to pathogens [49]. Additional insights into downy mildew resistance pathways include the role of *CsSGR*, a magnesium dechelatase gene that regulates chlorophyll degradation and whose mutation provides durable downy mildew resistance [60]. *CsLRK10L2*, identified as a candidate gene for sub-QTL *DM4.1.2*, acts as a DAMP oligogalacturonan receptor, and its loss-of-function mutation leads to necrosis in cucumber leaves and thus is also correlated with downy mildew [54].

Similarly, proteomic analysis identified various proteins differentially expressed between resistant and susceptible cucumber lines, with most involved in defense, cell rescue, and energy metabolism [64]. Zinc finger-homeodomain (ZHD) proteins, such as CsZHD1-3, CsZHD6, CsZHD8, and CsZHD10, act as plant-specific transcription factors responsive to downy mildew [65]. The CsIVP vasculature regulator (*Cucumis sativus* Irregular Vasculature Patterning) functions in integrating the programming of organ morphogenesis and was also correlated with downy mildew resistance [66]. While many candidate genes for downy mildew resistance have been identified, their roles need to be confirmed through overexpression or knockout studies in cucumber.

The genetic diversity within cucumber germplasm is crucial for breeding efforts. Accessions from various regions, especially those of Indian and Pakistani origins, have shown promising levels of resistance. For example, accession PI 197088 has been consistently identified as highly resistant in screening trials [50,54,57]. Exploring wild relatives and other Cucumis species may provide additional sources of resistance that are not present in cultivated varieties, enhancing the genetic base for breeding programs.

The recent advancements in cucumber genomics, including the sequencing of the cucumber genome, provide a valuable resource for identifying and utilizing resistance genes in breeding programs. The integration of molecular markers associated with resistance traits can enhance the efficiency of breeding efforts aimed at developing downy mildew-resistant cucumber varieties.

## 4. Management Strategies for Cucurbit Downy Mildew in Cucumber

Effective management of cucurbit downy mildew in cucumbers requires an integrated approach that combines cultural practices, chemical and biological control, and monitoring systems. By implementing these strategies together, farmers can reduce the severity of the disease and maintain healthy crop yields [15,29,33,41,67].

### 4.1. Cultural Practices

Cultural management techniques are the first line of defense against cucurbit downy mildew and play a crucial role in reducing the disease’s impact by limiting the conditions favorable for pathogen development [33,41,68,69]. Cucurbit downy mildew develops in moist, cool conditions, so increasing the space between plants and trellising cucumbers improves air circulation and reduces humidity around the foliage. Additionally, avoiding overhead irrigation and using drip irrigation can help prevent prolonged leaf wetness [23,24,41,70].

Implementing crop rotation with non-host plants also plays a very important role. Downy mildew pathogens can survive in crop residues and on alternate host species. Rotating cucumbers with non-cucurbit crops helps break the disease cycle by reducing the pathogen’s ability to overwinter and persist in the soil. From 2 to 3 years, rotation with non-cucurbit crops is recommended to minimize the risk of disease recurrence [70,71,72].

### 4.2. Resistant Cultivars

Genetic resistance is the most sustainable method of disease control. Breeding programs have focused on developing cucumber varieties that are resistant to cucurbit downy mildew. While complete resistance is rare, many cultivars exhibit partial resistance, which can reduce disease severity and the need for chemical treatments. Most popularly used cultivars of cucumber and cantaloupe, and, rarely, squash and pumpkin, have some level of this disease resistance bred into them [41,69,70,71].

### 4.3. Chemical Control

Fungicides are an essential tool in managing downy mildew, especially in regions prone to frequent outbreaks. However, fungicides should be used strategically to maximize their effectiveness and reduce the risk of pathogen resistance [33,73,74].

In areas with a history of severe cucurbit downy mildew outbreaks, regular fungicide sprays are necessary to protect cucumber crops. The timing of fungicide applications is key, with treatments often needed every 5–7 days, particularly during periods of favorable environmental conditions (cool, humid weather). A preventive spray program should begin early in the growing season before symptoms appear, as fungicides are generally more effective when applied before infection occurs [70,75,76]. Active ingredients of fungicides applied for cucurbit downy mildew include: cyazofamid, ametoctradin, ametoctradin + dimethomorph, propamocarb hydrochloride, azoxystrobin, azoxystrobin + oxathiapiprolin, copper (different salts) and sulfur [77]. In the USA, fungicides which contained oxathiapiprolin were the most effective against cucurbit downy mildew. Cyazofamid and propamocarb hydrochloride were the next most effective in suppressing disease severity [23,78,79].

Downy mildew pathogens can quickly develop resistance to fungicides if the same active ingredients are used repeatedly. That is why strobilurin fungicides (e.g., azoxystrobin) are no longer recommended against cucurbit downy mildew in the USA [70]. In Israel, certain strains of *P. cubensis* have become resistant to propamocarb hydrochloride and dimethomorph [23]. It is important to rotate between fungicides with different modes of action to prevent resistance development. Combining systemic fungicides, which provide longer-lasting protection, with contact fungicides can help achieve more effective disease control. Additionally, farmers should follow label recommendations to ensure proper usage and dosage [70,80].

### 4.4. Biological Control

The biological control strategy offers an alternative for managing downy mildew with less impact on the environment. This includes the application of plant extracts, bioactive ingredients isolated from plant sources or fish, bacteria cultures, biorational products, and plant resistance-inducing fungus.

Several medical plants have shown protection against cucumber downy mildew. The plant extract from *Glycyrrhiza glabra* showed 90% efficacy against downy mildew on cucumber plants [81]. Plant extracts from *G. glabra* and *Salvia officinalis*, as well as the bacterium culture of *Aneurinibacillus migulanus*, showed good efficacy of up to 80% against downy mildew in greenhouse-grown cucumbers [82]. Porsche et al. [83] demonstrated that leaf extract from *G. glabra* showed potent inhibition of the zoospore release and germination and the germ tube development of *P. cubensis* on both resistant and susceptible cucumber cultivars, reducing the infection rate by 73–96%. The active ingredients in the leaf extract from *G. glabra* included the antifungal polyphenols glabranin, pinocembrin, and licoflavanon, and a new compound, naringenin [83]. The extract of a medical plant, *Uvaria grandiflora*, and zeylenone isolated from this plant displayed antifungal activity [84]. In a pot experiment in the greenhouse, treatment of cucumber seedlings with a 5% microemulsion of *U. grandiflora* extract or zeylenone prior to *P. cubensis* inoculation effectively controlled downy mildew. The inhibition rates of *U. grandiflora* extract (82–100%) and zeylenone (70–99%) were comparable to the control treatment with the fungicide cyazofamid [84]. A secondary metabolite, named compound 3 (L-Pyroglutamic acid), isolated from the medical perennial herb plant *Disporopsis aspersa* (Hua) Engl. ex Diels was identified as the principal antifungal bioactive ingredient [85]. This compound exhibited efficient dural control effects on cucumber downy mildew. Spraying cucumber seedlings with compound 3 prior to *P. cubensis* inoculation gave a protection effect of 85.2%, higher than that of the fungicide control acrobat (82.8%). In contrast, treatment after *P. cubensis* inoculation showed an effect of 91.4%, higher than the fungicide control (85.9%) [85].

Pre-spraying the cucumber plants with garlic juice, which contained 50–1000 μg ml^−1^ allicin based on HPLC determination, reduced the severity of downy mildew by 50–100% [86]. Jhansirani et al. [87] demonstrated that extracts from garlic, clove, neem, tulsi, and pudina could significantly inhibit the sporangial germination of *P. cubensis* under in vitro conditions, among which garlic bulb extract showed maximum inhibition (71.42%), followed by clove oil (71.76%). Based on homology modeling and in silico docking analysis, the antimicrobial compounds from garlic (allyl acetate, allicin, and alliin) and clove (eugenol acetate and (*E*)-β-caryophyllene) displayed a high binding affinity with the RxLR effector (QNE4) and cytochrome c oxidase subunit 1 proteins of *P. cubensis* [87].

Diallyl disulfide (DADS), a major allelochemical of volatile organic compounds, is formed by the decomposition of allicin following the crushing of garlic. Treatment of the cucumber seedlings with synthesized DADS (laboratory-grade, purity 80%) significantly induced cucumber resistance to downy mildew [88]. Based on physiological indices and transcriptome analysis, the authors proposed a hypothetical mode of action of DADS. DADS treatment enhanced disease resistance by the activation of multifaceted defense machinery in leaves [88]. Dopamine, a water-soluble antioxidant, is one of the primary endogenous catecholamines in plants. Exogenous application of synthesized dopamine significantly decreased the severity of cucumber downy mildew by effectively alleviating the damage to cucumber photosynthetic and carbohydrate metabolism rates caused by the disease [89].

Glycoproteins are an environmentally safe alternative to fungicides [90]. Two glycoproteins, namely β-conglycinin isolated from soybean and p22 isolated from African catfish, showed significant antifungal activity against *P. cubensis*. In an open-field experiment, soybean β-conglycinin and catfish p22 glycoproteins reduced downy mildew disease severity to 20 and 10%, respectively, which was comparable to the ten highly efficient fungicide controls. Both glycoproteins showed complete protection for the new leaves against downy mildew. Microscopic examination of glycoprotein-treated leaves indicated that catfish p22 and soybean β-conglycinin could disrupt the integrity of the sporangial cell walls of *P. cubensis*, making the pathogen non-viable. Glycoproteins also triggered defense responses in cucumber plants, including elevated levels of antioxidant enzymes and phenolic content [90].

The antifungal protein isolated from the bacterium *Bacillus licheniformis* (isolate HS10) was identified as a carboxypeptidase which could significantly suppress *P. cubensis* on cucumber leaves [91]. Zheng et al. [92] identified new bacterial antagonists for downy mildew from different microenvironments of field-grown cucumber plants, e.g., *Bacillus pumilus* (isolate DS22), *B. licheniformis* (isolate HS10), *Enterobacter* sp. (isolate DP14), *Bacillus* sp. (isolate HP4), and *Stenotrophomonas maltophilia* (isolate DS57). In a greenhouse experiment, a foliar spray of bacterial cell suspension on cucumber plants prior to *P. cubensis* inoculation inhibited downy mildew with an efficacy of over 60%. In 2-year field trials, the efficacy of DP14, DS22, and HS10 for the control of naturally occurring downy mildew was 70.98 to 84.03% with foliar spray and 106.25 to 117.17% with foliar spray plus root drench, compared to the fungicide control propamocarb. DP14, HS10, and DS22 proved to be plant-growth-promoting rhizobacteria effective in controlling downy mildew in the field [92]. The bacterium *Streptomyces padanus* (strain PMS-702) has shown antifungal activity by producing a polyene macrolide antibiotic fungichromin [93]. In a greenhouse experiment, the application of PMS-702 cultural suspensions, prior to *P. cubensis* inoculation or co-application, significantly reduced downy mildew severity in cucumber compared to the water-treated control. PMS-702 cultural suspension significantly suppressed sporangial germination of *P. cubensis* based on microscopic examination [93]. The bacterium *Lysobacter enzymogenes* has shown a protective effect against downy mildew in cucumber [94]. The application of liquid culture suspensions of *L. enzymogenes* isolate, named LEC, at concentrations between 2.5 and 5%, on leaves of cucumber plants prior to *P. cubensis* inoculation was able to control the disease symptoms comparable to the fungicide control cuprozin progress treatment [94].

Three biorational products, i.e., microbial biopesticides Actinovate AG (active ingredient *Streptomyces lydicus*, a bacterium) and Serenade Soil (active ingredient *Bacillus subtilis*, a bacterium), and the biochemical biopesticide Regalia (active ingredient *Reynoutria sachalinesis* extract, a plant), can be applied in a rotational program with copper fungicide against downy mildew [95]. In a field experiment, treatments alternating copper fungicide with the three biorational products resulted in significantly lower cucumber downy mildew disease severity than in the nontreated control, which was not significantly different than the effect provided by the copper fungicide control champ WG [95].

*Trichoderma* fungi are recognized as inducers of plant resistance, among them *Trichoderma atroviride* (isolate TRS25). In open field conditions, both seed-coating with TRS25 and soil application of TRS25 with an organic carrier significantly reduced downy mildew and activated systemic defense responses in cucumber plants [96].

### 4.5. Monitoring

Monitoring crops and employing disease forecasting tools can significantly improve the timing and efficacy of management practices, allowing growers to respond quickly to early signs of cucurbit downy mildew or potential outbreaks [33,97].

Early detection of cucurbit downy mildew is critical for effective control. Farmers should inspect plants regularly for signs of infection, such as yellowing leaves and angular water-soaked lesions. These symptoms typically start on the older leaves and spread upward through the plant canopy. Prompt action at the first sign of infection can help prevent widespread crop loss [33,97].

### 4.6. Disease Forecasting Systems

Forecasting models use environmental data such as temperature, humidity, and rainfall to predict when conditions will be favorable for downy mildew outbreaks. By using disease forecasting systems, growers can anticipate when fungicide applications are most needed, improving the efficiency of chemical control and reducing unnecessary treatments. Several regions offer disease-tracking networks that issue alerts for cucurbit downy mildew, providing valuable information for growers to time their management interventions accurately [67,70].

## 5. Climate Change Impacts on Downy Mildew

Downy mildew represents a critical threat to cucumber production globally. The disease’s impact is especially concerning in the context of climate change, which modifies crucial environmental conditions that influence the pathogen’s development and spread. In the following section, we will discuss the epidemiology of downy mildew in cucumbers, with a particular focus on temperature sensitivity, humidity, altered weather patterns, and the evolution of the pathogen.

Downy mildew exhibits a marked sensitivity to temperature. Optimal conditions for the growth and spread of *P. cubensis* occur between 15 °C and 20 °C. At these moderate temperatures, the pathogen thrives, completing its life cycle rapidly and leading to widespread infections [15]. Warmer temperatures—particularly those above 25 °C—can suppress the pathogen’s development to some extent. However, while higher temperatures might slow the disease, they are often accompanied by changes in humidity and precipitation that can counteract this effect, facilitating disease outbreaks [28].

Humidity plays an especially important role, as the pathogen requires leaf wetness for sporangia to germinate and penetrate the host plant’s tissues. When temperatures hover in this optimal range, combined with high moisture levels, conditions become ideal for severe downy mildew outbreaks [28].

One of the most pronounced consequences of climate change is the increase in atmospheric humidity and the frequency of rainfall. These factors are critical to the establishment and spread of downy mildew. Relative humidity above 94% has been shown to be highly conducive to the pathogen’s infection process, as it significantly enhances the duration of leaf wetness, which is essential for germination. Climate change projections suggest that certain regions will experience more frequent instances of high humidity, further exacerbating the risk of disease outbreaks, particularly in cucumbers [15,28,98].

Extended periods of rainfall and increased moisture levels create an environment where cucumber crops remain wet for longer periods, providing a larger window of opportunity for *P. cubensis* to infect. This relationship between humidity and downy mildew risk is particularly concerning in areas where cucumbers are a primary agricultural product, as the disease can cause substantial yield losses under these conditions [98].

Shifting weather patterns due to climate change are expected to further influence the timing and intensity of downy mildew outbreaks. Historically, the pathogen tended to establish later in the growing season in northern regions. However, as climate patterns shift, it is likely that the pathogen will arrive earlier in the growing season in these areas, potentially exposing crops to downy mildew during more susceptible growth stages. This earlier arrival of the pathogen could significantly alter the disease management strategies that have been developed over decades of cultivation, necessitating new approaches to crop protection and disease forecasting. Furthermore, the increased frequency of extreme weather events, such as heavy rainfall and storms, may create more frequent and severe microclimates conducive to downy mildew proliferation, further complicating management practices [15,28].

Another dimension of the challenge posed by downy mildew under changing climatic conditions is the evolution of *P. cubensis* itself. In recent years, new strains of the pathogen have emerged that have overcome the resistance bred into many cucumber varieties. These evolved strains have proven capable of infecting resistant cultivars, leading to increased disease severity and yield losses, particularly in late-season crops when environmental conditions are more favorable for the pathogen [99].

The accelerated evolution of *P. cubensis* may be driven in part by the fluctuating environmental conditions brought about by climate change, as well as the widespread use of fungicides and resistant crop varieties. As temperatures, humidity, and rainfall patterns shift, the pathogen’s genetic diversity may increase, enabling it to adapt more effectively to new environments and host plants. This presents an additional layer of difficulty for disease management, as traditional resistance breeding strategies may not be as effective against newly adapted strains of the pathogen [100,101].

The impact of climate change on downy mildew in cucumbers is multifaceted, involving changes in temperature, humidity, and the pathogen’s evolution. Warmer temperatures may slow the pathogen’s development, but increased humidity and altered weather patterns may accelerate the spread of the disease. Additionally, the emergence of new, more virulent strains of *P. cubensis* poses a significant challenge to current management strategies. As climate change continues to alter the conditions under which cucumbers are grown, it is essential that future research and agricultural practices adapt to these new realities in order to mitigate the impacts of downy mildew.

## 6. New Technology for Early Warning and the Intelligent Control of Downy Mildew

Early detection of downy mildew infection before symptoms appear is of great importance for controlling the disease before extensive production loss occurs. Recently, computer vision technology, chlorophyll fluorescence imaging technology, machine learning methods, remote sensing tools, and intelligent control strategies have been used for combating downy mildew at an early stage [102,103,104,105].

The occurrence of downy mildew is closely related to the quantity of *P. cubensis* spores. Early detection of the spores is critical for the prevention and control of downy mildew before symptom development. Research advances in computer vision technology have made early diagnosis of plant pathogens possible. Qiao et al. [102] developed a deep learning model, YOLOv5s, for microscopic images of cucumber downy mildew causal agent spores. YOLOv5s is an improved real-time detection algorithm which effectively detects cucumber downy mildew causal agent spores based on a self-constructed microscopic image dataset. The application of YOLOv5s provided a basis for the early warning and control of cucumber downy mildew [102]. Chen et al. [103] demonstrated the use of chlorophyll fluorescence imaging technology, in combination with different machine learning methods and the convolutional neural network (CNN) transfer learning method, for the early diagnosis of greenhouse cucumber downy mildew in the seedling stage. The technique allowed for distinguishing the infected leaves from the healthy controls three days before symptom appearance [103]. Vatter et al. [104] demonstrated the remote sensing tool, leaf-clip sensor DUALEX, as a ready-to-use, low-cost device for real-time pre-symptomatic detection of downy mildew in cucumber plants grown under growth chamber conditions.

Liu et al. [105] proposed a hierarchical optimization strategy in the intelligent ecological control of the greenhouse downy mildew. The two-layer method aims to keep the optimum temperature for cucumber production but gives priority to reducing downy mildew infection risk. In the upper layer, the precise optimal control algorithm calculates and provides a suggested set-point when disease infection is simulated by the weather forecast and models to avoid ongoing infection. In the lower layer, a Proportional-Integral-Derivative (PID) controller keeps the optimum temperature for cucumber production, which considers energy conservation. The method combined greenhouse modeling, disease early warning, and environment control and considerably reduced downy mildew occurrence and the use of pesticides [105].

## 7. Future Directions and Challenges in Breeding for Downy Mildew Resistance

Cucumber downy mildew, caused by the pathogen *P. cubensis*, poses a significant threat to cucumber production worldwide, leading to severe yield losses. It is crucial to focus on developing effective breeding strategies in response to the emergence of new pathogen strains that can overcome existing resistance in commercial cultivars.

Current breeding, molecular, and genetic efforts have been mentioned in the previous sections of the current review article, but it is also worth highlighting that very extensive GWAS are underway to identify novel sources of resistance by screening various cucumber genome sequences [54,55,56,57,58]. The accurate translation of laboratory test results into agricultural systems can help minimize environmental pollution by reducing the use of pesticides and lowering cultivation costs. Cultivating downy mildew-resistant cultivars can lead to higher yields and increased profits for farmers [106].

Control of downy mildew in cucumber crops is also challenging due to the rapid evolution of *P. cubensis* strains. Based on the United States’ example, this phytopathogen has overcome the resistance of commercial cultivars [107]. In addition, the reliance on fungicides for managing downy mildew is not sustainable due to environmental concerns and the potential for resistance development in pathogens. Holdsworth et al. [107] undertook breeding studies to obtain new cucumber varieties with strengthened resistance. Cucumber cultivars “Ivory Queen” and “Marketmore 97”, with observed moderate resistance to downy mildew in the previous trials, were crossed, and the DMR-NY264 breeding line exhibited a high level of resistance and good yields [107]. Developing new resistant cultivars through breeding programs may also consider using other well-known cucumber accessions with acknowledged downy mildew resistance, such as PI 197088. On the other hand, expanding genetic diversity by exploring wild relatives and lesser-known cultivars could enhance resistance breeding efforts [52].

In light of the future developments in breeding and research on cucumber and other cucurbits, it is important to explore the potential of cutting-edge genomic tools. Among the major techniques used in plant breeding, cross-breeding and mutational breeding are time- and labor-intensive and may generate cultivars with undesirable features along with the resistant gene due to linkage drag or unwanted genetic changes [108,109,110,111,112,113]. Releasing the resistant cultivars obtained through transgenic breeding may require a long and costly regulatory evaluation process [111,112,113,114]. Advanced genomic techniques such as CRISPR/Cas9 gene editing could facilitate the precise modification of cucumber genomes to enhance downy mildew resistance. This approach enables specific changes to improve resistance while preserving other agronomic traits. Many local varieties with good taste and nutritional qualities, but which remain susceptible to the pathogen, could be the targets for genome editing to increase disease resistance in such economically highly valuable crop genotypes. To date, the scientific literature has described two approaches to modifying cucumbers to increase their resistance to downy mildew. In the first one, in the study of Yin et al. [115], the PR-2d promoter/*uidA* gene construct was introduced into the cucumber genome via *Agrobacterium tumefaciens*-mediated transformation. The PR-2d promoter was fused to the *uidA* gene (β-glucuronidase gene) from tobacco (*Nicotiana tabacum*) and introduced into a highly inbred line of *C. sativus* L. cv. Borszczagowski. Inoculation with fungal pathogens, i.e., *P. cubensis*, increased the concentrations of endogenous salicylic acid and salicylic acid glucoside in transgenic cucumber leaves, where these compounds were found to have a significant role in enhancing plant defense mechanisms against various biotic and abiotic stresses [115]. Furthermore, Dong et al. [116] described a significant milestone in the research on interactions between cucumbers and downy mildew in 2023. This study was the first confirmed use of CRISPR/Cas9 in cucumbers that effectively boosted their resistance to downy mildew. The authors applied the genome-wide association study and found a single nucleotide polymorphism (SNP) in the *STAYGREEN* (*CsSGR*) coding region at the gLTT5.1 locus related to the low-temperature tolerance. Interestingly, knockout mutants of *CsSGR* generated by CRISPR/Cas9 exhibited enhanced tolerance to the various biotic and abiotic stresses, including *P. cubensis* infection, low temperature, salinity, and water deficit [116].

Moreover, utilizing gene-editing technologies like CRISPR-Cas9 is crucial for uncovering the exact roles of different genes in cucumber growth, development, and stress responses. Many studies have used CRISPR/Cas9 to examine the effects of specific genes on downy mildew disease. For example, besides the aforementioned study in cucumbers, it was found that knocking out the *DMR6* gene in sweet basil (*Ocimum basilicum*) led to resistance against *Peronospora belbahrii* (basil downy mildew) [117]. Additionally, the loss of function in pathogenesis-related 4 (PR4) protein has been shown to decrease downy mildew resistance in grapevines (*Vitis vinifera* L.) caused by the oomycete *Plasmopara viticola* [118]. Furthermore, the CRISPR/Cas9 technology enables the development of transgene-free plant resistance, which might be better accepted by public opinion compared to genetically modified organisms (GMOs) [117].

## 8. Conclusions

The genetic and molecular study of downy mildew resistance in cucumbers is crucial for developing durable, high-performing resistant cultivars, which are key to sustainable disease management in cucurbit production. The identification of major resistance genes and QTLs, combined with modern molecular breeding tools such as marker-assisted selection and genomic selection, offers significant potential for accelerating breeding efforts. However, the challenge of pathogen evolution, including the emergence of new virulent races, highlights the importance of ongoing genetic research and the integration of multiple resistance mechanisms. By combining traditional and modern breeding approaches, along with a deeper understanding of the molecular mechanisms underlying resistance, scientists can develop long-term strategies for managing downy mildew in cucumbers. This will contribute to reduced fungicide application, increased crop productivity, and overall sustainability in cucurbitaceous crop production systems. In summary, the genetic basis of downy mildew resistance in cucumbers is complex and involves multiple genes and QTLs. Continued research into the genetic mechanisms underlying this resistance will facilitate the development of more resistant cucumber cultivars, which is essential for sustainable cucumber production in the face of ongoing disease challenges.

## Data Availability

No new data were created or analyzed in this study. Data sharing is not applicable to this article.
